# The Indexical Voice: Communication of Personal States and Traits in Humans and Other Primates

**DOI:** 10.3389/fpsyg.2021.651108

**Published:** 2021-04-15

**Authors:** John L. Locke

**Affiliations:** Lehman College, City University of New York, New York, NY, United Sates

**Keywords:** cues, signals, alarm calls, close calls, grooming, lip-smacking, small talk

## Abstract

Many studies of primate vocalization have been undertaken to improve our understanding of the evolution of language. Perhaps, for this reason, investigators have focused on calls that were thought to carry symbolic information about the environment. Here I suggest that even if these calls were in fact symbolic, there were independent reasons to question this approach in the first place. I begin by asking what kind of communication system would satisfy a species’ biological needs. For example, where animals benefit from living in large groups, I ask how members would need to communicate to keep their groups from fragmenting. In this context, I discuss the role of social grooming and “close calls,” including lip-smacking and grunting. Parallels exist in human societies, where information is exchanged about all kinds of things, often less about the nominal topic than the communicants themselves. This sort of indexical (or personal) information is vital to group living, which presupposes the ability to tolerate, relate to, and interact constructively with other individuals. Making indexical communication the focus of comparative research encourages consideration of somatic and behavioral cues that facilitate relationships and social benefits, including cooperation and collaboration. There is ample room here for a different and potentially more fruitful approach to communication in humans and other primates, one that focuses on personal appraisals, based on cues originating with individuals, rather than signals excited by environmental events.

## Introduction

“We do not really know what a man is saying until we know who he is and to whom he is speaking.” F. G Bailey (1972), Gifts and Poison, 1972

In a book published in 1944, the physicist, Erwin Schrodinger, pointed out that the body of an organism survives by ingesting “negative entropy.” Few knew what that meant, but as the psychologist, George Miller would later write, there are mathematical similarities between entropy and information. If the mind “survives by ingesting information,” as he claimed, “all higher organisms are informavores” (Miller, 1983, p. 111).

Clearly, that designation includes humans. In the 1970s, sociologists began to use terms like “Information Age” and “Information Society” in reference to times and places where mediated communication systems were facilitating the flow of messages between individuals. But this information had an unusual property for humans and other mammals: neither the sender nor the receiver was physically present, nor did they necessarily know or care about each other. This arrangement may have reinforced a disembodied perspective on human communication.

Prior to the evolution of symbolic communication, humans would have regularly inferred each other’s states and traits, purely from observation, as the other primates do. But in our species there was a tilt from individuals to messages, which may have diminished aspects of personal information, including the identity and nature of the participants. Here I suggest that this cultural effect may have caused us to think that humans and other primates are less alike than they actually are.

## Information

For many decades, theories of human communication reflected the ideas of two signal engineers, Claude Shannon and Warren Weaver, who famously declared that if speakers tell listeners something they already know, no information passes between them (Shannon and Weaver, 1948). In this stripped-down view of communication, little thought was given to the possibility that speakers might communicate something besides the nominal topic, e.g., a willingness to share the material that they’re expressing; a presumption that the listener doesn’t know the thing that they’re saying; or a belief that the listener would find it interesting.

Shannon and Weaver may have offered a reasonable interpretation of information theory, as formulated, but in real life people are often drawn to individuals with whom they share knowledge. It can be pleasurable, even exciting, to discover that friends know many of the same things that we do. Shared knowledge represents a form of inter-personal similarity which, like other instances of homophily, tends to promote affiliation (Launay and Dunbar, 2015).

In our lives, shared knowledge also lays the social and psychological groundwork for cooperation—an important issue to which we’ll return later—but in a corporate context, working groups are often set up such that each member has something unique to contribute. The expectation is that team members will naturally pool their information, but the expectation is often unfulfilled due to the fact that a stronger disposition—to discuss common knowledge—gets in the way (Wittenbaum, 2000).

If the transmission of information is an important function of speech, then this should be revealed in various kinds of behavioral tests. For example, we might expect to find that novel information is more carefully articulated than familiar information, or is less guessable in the presence of noise, or—perhaps the best test—that speakers go out of their way to avoid saying anything that listeners might find ambiguous.

## Ambiguity

If the transmission of information was the primary purpose of speaking, ambiguity would pose a serious threat to communication, but it’s not clear that it does. Many of the things that people say are structurally ambiguous (e.g., Amy likes intelligent men and dogs), indicating that speakers rarely avoid this property, even when the possibilities for doing so are readily available (Ferreira and Dell, 2000; Ferreira et al., 2005; Haywood et al., 2005). Steven Pinker and his colleagues have pointed out that speakers not only tolerate ambiguity, they actively seek it. That’s also true of indirect speech, e.g., where one diner asks another, “Can you reach the salt?” but gets the salt instead of an answer. These kinds of formulations are “inefficient, vulnerable to misunderstanding, and seemingly unnecessary” but are used universally (Pinker et al., 2008).

What I will emphasize here is that there are social factors that qualify, even mitigate, the value of semantic precision. In the first part of this paper, I venture into an empirical arena that is familiar to most primatologists, the transmission of information about environmental opportunities and dangers—specifically predators—raising questions about the relevance of this issue to its stated or implied context: the evolution of language. Then, I turn my attention to what I think of as “first principles,” asking what kinds of communications would have been required in evolutionary history, given the social structure of humans and some primate species, and compare this to what actually exists. Here, I look at more promising areas of overlap among humans and other primates, specifically the socially functional but less semantically loaded communication registers related to “small talk” and, among the so-called “close calls,” grunts and the lip-smacking associated with social grooming. Finally, I examine a wholly different class of information, one that pertains to the nature of the individual. I call this information “indexical,” and suggest that human and non-human primates are remarkably similar when it comes to this class of inter-individual communication. Here I distinguish between information that is sent in the form of signals from information that is emitted in the form of cues. But first I will discuss something quite different, partly to make my point: the alarm calls of vervet monkeys.

## Alarm Calls

Some of the more exciting research on primate communication was done in East Africa on vervet monkeys, who were thought to issue a distinctive call in response to each of three classes of predators—snakes, eagles, and leopards. It was thought that these alarm calls warned other members of the group to take evasive action, and since vervet calls do not resemble the calls of the predators themselves, they were thought to be symbolic, much as words are.

If one were looking for informative uses of the voice in primates, these calls might seem to have qualified on several counts, and the initial reports were positive. The primary investigators, Robert Seyfarth and his colleagues, concluded that vervet monkeys “give different alarm calls to different predators. Recordings of the alarms played back when predators were absent caused the monkeys to run into trees for leopard alarms, look up for eagle alarms, and look down for snake alarms.” (Seyfarth et al., 1980, p. 801). They even found that predator classification improved with age and experience.

The excitement was palpable. “Here,” as several primatologists would later write, “there was evidence of language-like communication in a monkey, with the promise of similarly human-like cognitive complexity. The implications for language evolution were tantalizing.” (Rendall and Owren, 2013, p. 153) If any primate vocalizations could be construed as meaningful, these calls would seem to be the closest thing to human speech that had been discovered to date. For, even if they were issued under conditions of extreme agitation and fear, alarm calls seemed to inform others in ways that appeared to be *verifiable*, surely a desirable criterion if these calls were certifiably to qualify as meaningful. But this attribute comes at a price, one that I will suggest is unacceptable. For, in search of signals whose meaning could be ascertained, researchers tended to avoid social vocalizations, which produce important but less discrete or observable responses (see descriptions in Silk, 2002; Silk et al., 2013).

If alarm calls were ambiguous, that is, if a call, like a yell, only meant that others should look out for *something*, I doubt that they would have excited much empirical attention, and in the end that seems to have been the result. Julia Fischer and her colleagues concluded that little in the way of supporting evidence was actually obtained or reported (Fischer et al., 2015; Fischer, 2017; also see Price et al., 2015). Moreover, vervets also gave similar sounding calls in other aggressive contexts.

There’s irony here. While primatologists were trying to demonstrate that monkeys *mean something* with their calls, some linguists were demonstrating that many human utterances *mean little*—at least literally—given canned phrases such as *biting the hand that feeds you, in a nutshell, at the end of his rope, in the nick of time*, and *quit cold turkey* (Sidtis and Sidtis, 2018). So even if it was exciting to think that vervet monkeys *might* be using distinctive vocalizations to inform each other, there were issues to be considered if language was to be considered creative or generative as well as informative.

## But, What If It Were True?

The hypothesis of Seyfarth and his colleagues was ultimately disconfirmed, but what if agitated and frightened monkeys had verifiably transmitted predator-specific information? How would a handful of innate signals by individuals who were agitated or frightened, issued more or less reflexively, help us to understand language, a complex, unlearned medium that is used flexibly and socially, sometimes in jest? Fischer (2021) has suggested that vervet alarm calls may be innate. If that’s true, can we say that vervets *do* something in order to inform each other, or merely *experience* events by reacting in an audible way?

Receptive components of primate vocalizations might even be innately present in another class of primates: humans. Thirty years ago, a team in Finland asked naïve humans to classify the affective content of macaque vocalizations that had been recorded when the animals were in situations associated with aggression and fear, sexual excitement, dominance and several other emotions. The listeners were extremely accurate in classifying the affective qualities of these vocalizations that they had never heard before (Leinonen et al., 1991).

Before moving on, I should restate my reason for bringing up alarm calls. It was not to add my voice, superfluously, to the conclusion that they may not be truly symbolic. It’s to make a different sort of claim altogether: that studies of shrieking in a state of extreme agitation were never an appropriate way to find common ground with speech or language—cognitively, neurologically, or socially—whether the shrieks were precipitated by anything in the environment or not.

## First Principles

When approaching the communication system of any species, it helps to begin by considering first principles, that is, the reasons why members of that species would need to communicate *at all*; and what kinds of information, and in what form, its members would benefit from exchanging. What kind of communications would have improved the lot of our evolutionary ancestors? If they needed to harmonize their interests, and to trust and cooperate with each other—which now seems obvious—they presumably required a means of communicating that would enable them to achieve these benefits. What kind of communication system would facilitate these objectives?

There are two simple questions here: what do members of a particular species do *in order to* communicate, or that *happens to* communicate, and what, given things we know about members of that species, would they be expected to do? Let’s begin by reviewing some social facts, ones that may have played a role in evolution. The first relates to the effect of group size on external vigilance and within-group attention. When primate groups were small, there was considerable risk of predation, and the voice was needed to warn other members of the group whenever predators were detected (Port et al., 2020). Our distant ancestors kept an eye on the periphery of their tiny camps, where predators lay waiting.

When groups enlarged, members spent less time *looking for* predators and more time *looking at* members of their own group, in search of individuals with whom they might cooperate and collaborate (Locke, 2005). Doing so would have been essential, for if larger groups increased competition for resources, as is widely assumed, members would have needed innate mechanisms or strategies to keep their groups from splitting up.

Work by Robin Dunbar suggests that evolutionary increases in the size of primate groups produced new levels of social complexity, challenging and ultimately enhancing the interpretive capacity of the social brain and the use of vocalization to service relationships (Dunbar, 1992, 1993, 1998, 2009; also see Gustison et al., 2012; Roberts and Roberts, 2019). These changes in neural and vocal complexity may have been as adaptive as alarm calls. For if enlargement of groups helped to foil or reduce predation, other adaptations would have been needed to ensure that groups *retained their membership*. One of these may have been tonic communication, a call-response tactic that, according to Wolfgang Schleidt (1973, 1977), helps to keep groups together. There may also have been pressures to ramp up the analytical and interpretive abilities of receivers.

## Close Calls

Many animals live in stable social groups and their fitness, according to Joan Silk and her colleagues, “depends at least in part on the outcome of their interactions with other group members.” (Silk et al., 2013, p. 213). The success of these interactions is affected, in part, by the animals’ use of “close calls” (Harcourt et al., 1993). Several classes of close calls, including lip- or tongue-smacking, grunts, and girneys, have been identified. As their name implies, these sounds are used by familiar individuals at close range, and with significant social consequences.

Any boost in primate sociality presupposes neural commitments to a mode of communication that would facilitate the evaluation of individuals for a variety of short- and long-term relationships. In a study of socialization and vocal behavior, McComb and Semple (2005) analyzed reports on forty-two different primate species, finding strong relationships among the size of primate groups, the time devoted to grooming, and size of vocal repertoires. Based on these findings, McComb and Semple suggested that a greater number of different vocalizations may be needed for animals to navigate complex networks of social relationships in primate societies.

## Relating

Speech enables people to perform in a public way, a behavior that particularly appeals to adult males in their quest for power or status and mating opportunities (Locke, 2001, 2011). But is the primary purpose of speech to perform, to inform, or to relate? In *The Tongues of Man*, the English phonetician, John Rupert Firth, wrote at length about the organs of speaking, including the tongue, lips, and jaw, but then shifted his attention to the “organs of talking.” These, Firth said, “are at least two *normally associated human beings*.” (Firth, 1937, p. 152).

One senses that Firth was onto something with his use of the word, *talking*, for it—like chatting—implies something about the social applications of speech. But what did Firth mean by talking? He didn’t say. Nor did Darwin when he made a similar reference. He had heard naturalists remark that social animals who habitually use their vocal organs “as a means of intercommunication, use them *on other occasions* much more freely than other animals.” (Darwin, 1872, p. 84, italics mine) What he meant by “other occasions” is unclear but, as I will discuss shortly, primate vocalizations are not limited to calls, nor do they invariably convey information about the physical environment.

If we look at the way people express themselves when relating to friends, we are likely to see something that is grammatically-simple, colloquial, predictable, redundant, structurally incomplete, and semantically imprecise. In fact, as I suggested earlier, much of it is not all that linguistic. If one wanted to study recursion, which some linguists take to be the hallmark of grammar, it’s not clear that everyday conversational speech would be the best place to find it (Hauser et al., 2002). But if we were interested in comparing humans and other primates on the tendency to relate, the most suitable behavior would probably be manual grooming.

## Social Grooming

Manual grooming—sorting through the fur of an animal in search of parasites—may appear to be a nutritive process since groomers consume the yield, which contains protein. But grooming is primarily a social process. It tends to work upwardly, lower ranking animals being more likely to groom those of higher rank than the converse, and it acts like a favor (Cheney, 1977; Cheney and Seyfarth, 1990). de Waal (1997) has reported that animals are more likely to share food with another animal if they had previously groomed him than if they had not done so.

Grooming figures prominently into the formation and maintenance of social and cooperative relationships, but animals have other things to do besides groom. If grooming is performed dyadically, animals may be unable to maintain a satisfactory number of relationships. Dunbar (1993) proposed that polyadic conversations, which allow access to several social partners simultaneously, evolved as a form of social grooming to circumvent this time constraint. In non-human primates, it appears that polyadic grooming enables animals to maintain weak social relationships with many partners (Girrard-Buttoz et al., 2020).

Dunbar also suggested a second mechanism for the expansion of social relationships, what has come to be known as grooming-at-a-distance or, since that’s physically impossible, vocal grooming. Malgorzata Arlet and her colleagues compared the rate of contact call exchanges between the females in two captive groups of Japanese macaques. They found a positive relationship between the time devoted to grooming by two females and the frequency with which they exchanged calls. Their results were consistent with predictions of the social bonding hypothesis, which holds that vocal exchanges can be interpreted as grooming-at-a-distance (Arlet et al., 2015; also see Kulahci et al., 2015).

## Lip-Smacking and Grunting

Nearly a century ago, English zoologist Solly Zuckerman observed a colony of baboons and noticed “rhythmical lip, tongue, and jaw movements that usually accompany *friendly advances* between two animals, and that continue throughout the process of grooming.” (Zuckerman, 1932, italics mine).

Forty years passed before anyone discussed the movements that were actually involved in lip-smacking. Then, one primatologist commented that “the actual smacking noise appears to be made by the *tongue* breaking contact with the roof of the mouth and/or upper lip or row of teeth, rather than by the lips themselves parting.” (Redican, 1975, italics mine). But, for some reason, this activity came to be known as *lip-*smacking. Later, others measured and commented on lip-smacking’s physical characteristics (e.g., Ghazanfar et al., 2012; Pereira et al., 2020), but it was the friendly advances that made lip-smacking interesting from a social standpoint.

The same goes for grunting, which is often used to signal peaceful intentions (Silk, 2002). In a study of wild Guinea baboons, Lauriane Faraut and her colleagues found that when approaching baboons grunted, they were more likely to interact in an affiliative fashion and less likely to displace the partner (Faraut et al., 2019). One could include other such studies here, but the point is that primates learn things about each other when they grunt and groom. “We are reasonably certain,” wrote Joan Silk, “that monkeys make use of *information* derived from their own interactions with other group members to regulate their social relationships.” (Silk, 2002, p. 153, my italics).

If there are classes of primate vocalization that convey information and *mean*, in effect, that an animal intends to be friendly—and on the basis of these vocalizations animals are able to form and maintain relationships—we are surely entitled to ask what is meant when we humans greet others (Laver, 1975).

## What Do “We” Mean?

When we hear a primate smack his lips, we don’t ask what the individual smacks *mean*. We’re aware that when it comes to semantics, it’s not this smack or that smack, it’s the act of *smacking*. “The medium,” we might say, reminiscent of the 1970s media guru, Marshall McLuhan, “is the message.” Since Austin (1962) and, before him, Peirce (1878), it has been clear that the medium—material that exposes the speaker’s intention, whether it is, e.g., to praise, accuse, or belittle—can be a more important feature of a conversation than any of the words that are used.

In Zuckerberg’s work, the animals that were advancing in a friendly way were Hamadryas baboons, but he could almost as easily have described a reunion, in our own species, of two friends who have just reconnected after a period of separation (Laver, 1975). It is that sort of friendly interaction that led social anthropologist, Bronislaw Malinowski, to propose the term, “phatic communion,” for a sense of connection achieved by familiar individuals when speaking. Phatic communion, he said “serves to establish *bonds of personal union* between people brought together by the mere need of companionship and does not serve any purpose of communicating ideas.” He added that “It is *only in certain very special uses among a civilized community*, *and only in its highest uses that language is employed to frame and express thoughts”* (Malinowski, 1923, p. 316).

Malinowski’s view was inspired by his work in a small-scale society, but it has been observed in analyses of ordinary speech in more progressive cultures. One example was supplied by an American couple that allowed themselves to be recorded while on holiday. An analysis of nearly two thousand messages spoken by the couple revealed that fully three-fourths of their utterances were comments that involved *no* facts or other concrete information (Soskin and John, 1963).

The search for information in primate calls was understandable, given the desire to see them as referential, but much of human *speech* doesn’t “mean” much, word for word, compared to the fact that the *speaker* has chosen to verbalize and has done so in a friendly manner, which may mean that he intends no harm, would like to interact, and is open to friendship. That’s a huge message, even if it might seem to be small.

## “Small Talk”

A century ago, in a short essay called “Small-Talk,” an English writer described a semantically empty type of speech that is undertaken “not for the sake of saying something, but for the sake of saying anything” (Friedlaender, 1922). Forty-five years later, ethologist Desmond Morris offered a name for the sort of social speech the writer described. In *The Naked Ape*, he referred to “the *meaningless*, polite chatter of social occasions, the “nice weather we are having” or “have you read any good books lately” form of talking.” The purpose of this sort of chatter, he said, is “to reinforce the greeting smile and to maintain the social togetherness.” It *“is not concerned with the exchange of important ideas or information*.” (Morris, 1967, p. 204, italics mine). He called this “meaningless” chatter, “grooming talking.”

Reminiscent of Morris, philosopher Charles Taylor asked his readers to imagine that they were traveling with him on a train that is moving through a southern country. At some point, he says to a fellow passenger, “Whew, it’s hot.” This, he recognizes, “doesn’t tell you anything you didn’t know; neither that it is hot, nor that I find it so. Both these facts were plain to you before. Nor were they beyond your power to formulate; you probably already had formulated them.”

What, then, was accomplished by this exchange? What it did, Taylor said, was “to create a rapport between us, the kind of thing which comes about when we do what we call striking up a conversation. Previously I knew that you were hot, and you knew that I was hot, and I knew that you must know that I knew that. But now it is out there as a fact *between us.”* (Taylor, 1985, p. 273, italics mine).

Whether Taylor’s traveler knew it or not, he was laying the groundwork for something else: cooperation. Suppose, for example, he recognized at some point that he needed to leave his seat for a few minutes. If so, he might feel comfortable asking his seat-mate to keep an eye on his things while he was gone, something he might feel less comfortable doing if they had not yet “broken the ice.”

What few seem to have recognized is what might be occurring *during* small talk that is so cognitively undemanding that it can be processed by listeners while evaluating the speaker, which may be the primary purpose of the interaction, not the transmission of verbal information. Quiet conversation grants them the proximity and time required for the evaluation of weak somatic cues. A meta-analytic study by Balliet (2010) suggests that the mere act of communication enhances cooperation, especially in large groups of people. Which is interesting in light of primate work indicating that lip-smacking facilitates cooperation in wild chimpanzees (Fedurek et al., 2015).

There’s one final point to be added here, one that is no less important. When we hear a message that has little semantic content, it is likely that the message is not the information that the speaker intended to convey, but the speaker himself: what he is like at the moment and may continue to be like in the future.

## Indexicality

Information *is* conveyed by the traveler in Taylor’s anecdote, but it is *about the traveler himself*. From it, we may guess that he is, in the present situation, bored, lonely, open to interaction, feeling sociable, and a great many other things; and we may also infer that he is generally a friendly person. Given the length of the journey, one or more of these things may have been exactly what he needed to convey and his fellow passenger needed to know. The most important thing group-living individuals can know about others is *who they are*, that is, which of various individuals they happen to be, and what physical and behavioral characteristics they happen to possess.

In quiet conversations among familiars, what do utterances mean? Obviously, it depends on the topic, or does it? I suggest that what they mean, in the broadest and simplest sense is THE SPEAKER, as he was before and during the interaction. I suggest that the most basic and useful information that is orally communicated by humans (irrespective of culture) and other primates is personal, including information about behavioral dispositions in the moment, ones linked to transient physiological and emotional states, and reactions to observers, and stable tendencies to aggress, relate, or cooperate.

Which brings me to my main point: what would our group-living ancestors have needed to learn about each other? I will use the term *indexical* to represent characteristics of individuals who are emitting or sending information that is about them, whether it is in the form of transient physiological activity or emotion, or the expression of relatively stable features including temperament and personality. Fifty years ago, English phonetician David Abercrombie (1967, p. 9) used that term in reference to variations in a person’s speech that “come and go according to his physical or mental state” (also see Peirce, 1878). For Abercombie, examples of physical and emotional states included excitement and nervousness, which directly affect the operation of the vocal organs, therefore, the voice and speech of the individual, producing “affective indices.” “When a person speaks,” wrote another British phonetician, John Laver, “he reveals often very detailed indexical information about his personal characteristics of regional origin, social status, personality, age, sex, state of health, mood, and a good deal more.” (Laver, p. 221).

These phoneticians seized upon unintended variations of voice and speech precisely because they are unintended, therefore, like a nuisance variable, not properly considered a property of the language that was being described. Not being semantically critical, unintended material is likely to carry the most information about the speaker.

Whether speaking or not, Rendall and Owren (2013) have pointed out that we all communicate by way of a *biological code* which is neither arbitrary nor, necessarily, learned. This code carries information “about relevant social or physical characteristics of signalers such as their age, sex, body size, individual identity, emotional state or physical condition.” (Rendall and Owren, 2013, p. 162). What they chose not to discuss is their value in linguistic communication, which may be to amplify or even to negate the literal meaning of any words that are embedded in the same acoustic stream.

Research now indicates that human listeners—even those who have never studied primates—are able to discriminate individual monkeys from their coos and screams, naturally and without training, much as they discriminate between members of our own species from their speech, even if limited to isolated vowel sounds (Owren and Rendall, 2003).

Earlier, I asked if vervets provide others with information or were merely experiencing something in a way that excited vocal behaviors that are audible to nearby others. Years ago, sociologist Irving Goffman (1959) offered two simple labels for the kinds of information that people exchange in their interactions. Some of it, he wrote, is “given,” by which he meant material, typically in the form of words and gestures that we send to others deliberately. It is often self-serving and therefore may be unreliable. The donors are consciously aware that they are donating something and may even be able to anticipate its effect on recipients.

To describe the other kind of information that people communicate, Goffman used the term “given off.” This information becomes available to others merely because they happen to be close enough to absorb it. People in sensory range discover things about us whether we want them to or not. Some of the cues are expressed by glands that emit chemicals into the atmosphere, announcing changes in physiological and emotional states. Others are leaked earlier in life, often under genetic influence, inscribing on the face and body lasting messages about the occupant.

## Cues and Signals

In the evolution of vocal communication, it is important to distinguish between *cues*, the information that is given off, and *signals*, the information that is given. A cue to some physical or behavioral feature that is informative may occur as an *emission*. Examples include a loud voice, which may imply health or physical strength (Sell et al., 2010). But cues that are emitted can evolve into signals that are sent if their reproductive value is actively displayed or exaggerated (Maynard Smith and Harper, 1995; Fitch and Reby, 2001).

The existence of these cues may have contributed to the development of appraisal mechanisms that enabled our ancestors to cooperate selectively with individuals that had something to offer. I will suggest here that some primate species evolved ways to interpret the cues to transient states and stable traits, enabling them to select suitable partners for cooperative relationships.

In the last 20 years, a great deal has been learned about the physical cues to various personal qualities in humans. While primatologists have wondered whether non-human primates were capable of symbolic behavior, social psychologists have been asking how much humans learn about each other from various cues, some auditory or visual, others, thanks to recent and ongoing research, olfactory. It is not clear, at the moment, what all or even most cues to personal qualities are in primates, though some in the acoustic, chemical, visual, and tactile modalities have been identified (Moreira et al., 2013).

### States

There are two broad classes of information that are given, or given off, by the communicants. One is whatever the individuals are experiencing in the moment, given the situation in which they find themselves. Much of this information, naturally, is emotional and is properly regarded as “affective” (Rendall and Owren, 2013). The rest of it is physiological, including changing levels of stress or sexual readiness. Both kinds of information, emotional and physiological, qualify as transient states.

### Traits

Traits include whatever physical cues enable others to identify the caller or speaker as the individual that he or she uniquely is and a second class of information that includes what these individuals are like, that is, how they are best described in terms of personality or temperament and, in humans, character. These relatively *stable traits* are unusually important, for they enable others to predict future behaviors, therefore to approach or avoid individuals for mating or other cooperative activities in the future.

If personal traits seem particularly relevant to humans, it is important to recognize that non-human primates also emit cues to a number of physical and behavioral characteristics, ones that, for example, predict dominance, aggressiveness, and other behavioral dispositions. Like humans, the other primates also have histories, reputations, and essential qualities that are associated with temperament and personality.

We all know that humans have personal qualities, including temperaments, that appear early in development and continue well into adult life (Tang et al., 2020). In our species, these enduring traits are particularly important, given the need to make social and reproductive choices that have long-term consequences. What is the equivalent, if there is one, in other primate species? Whether we use the word “personalities” or not, it is recognized that some chimpanzees are gregarious, others bold or aggressive; some are risk-takers, open to new experiences, others are more introverted or shy. Significantly, some primates are known to be socially tolerant, a prerequisite to life in large groups in general and to cooperation specifically (Hare et al., 2007; Cieri et al., 2014; also Melis et al., 2006).

In a meta-analysis, Freeman and Gosling (2010) discovered a set of personality variables that had been reported in several hundred studies of primates, mostly adult chimpanzees and macaques. The most frequently shared traits were fearfulness, dominance, and confidence or aggressiveness, along with irritability, sociability, playfulness, and activity. Patrick Tkaczynski and his colleagues have reported that multiple types of social behavior were repeatable over the long term—up to 19 years—in wild chimpanzees. They concluded that “chimpanzees living in natural ecological settings have relatively stable long-term social phenotypes over years that may be independent of life-history or reproductive strategies.” Their results, they said, “add to the growing body of the literature suggesting consistent individual differences in social tendencies are more likely the rule rather than the exception in group-living animals” (Tkaczynski et al., 2020, p. 1).

## Emerging Points of Agreement and Continuity

Though it has passed largely unrecognized, points of possible agreement between human and non-human primates have been quietly developing where indexical attributes are concerned. While primate research was exposing a gulf between human language and primate calls, a separate body of evidence was steadily exposing similarities between these species. For the fact is, when primatologists were looking for the seeds of speech, therefore language, evolutionary psychologists were looking for—and finding—a number of evolutionary antecedents to human communication.

### Indexical Vocalization

Humans communicate a great deal of information about their states and traits. Some travels vocally. For example, there is evidence that men with low-pitched voices have more testosterone than other men, and are thought by female listeners to be more dominant and attractive (Collins, 2000; Feinberg et al., 2005; Puts et al., 2006), especially when their voice is heard in a courtship or mating context (Apicella and Feinberg, 2009; Little et al., 2011). This preference is stronger when women are in the fertile phase of their ovulatory cycle when estrogen levels are unusually high (Puts, 2005; Feinberg et al., 2006).

The human voice may be a reproductive cue when it varies with sex hormone levels, but it can also be appropriated for use as a reproductive signal. Lower vocal pitch predicts the mating success of males (Apicella et al., 2007). It has been reported that men lower their pitch, and that women may raise theirs, in a contrived mating context (Puts et al., 2006; Fraccaro et al., 2011). But, not everything is vocal.

### The Indexical Face

In a reincarnation of classic physiognomy, it’s been found that some personal traits can be accurately inferred from the dimensions of one’s face—especially in men. Its the predictive value of a ratio—between bizygomatic width, that is, the lateral distance between left and right cheekbones (the zygions), and upper face height, that is, the vertical distance between the upper lip and the superior surface of the eyes. The typical ratios for adult females and males are about 1.80 and 1.86, respectively (Carré and McCormick, 2008). This difference is thought to reflect the fact that in adolescence, when the face usually elongates, a surge of testosterone tends to suppress this lengthening process in males (Ursi et al., 1993; Verdonck et al., 1999; Bulygina et al., 2006; Lefevre et al., 2013). The result is a face that is wider for its height than the average female face, with relatively larger cheekbones (Weston et al., 2004).

In humans, facial width and direct measures of testosterone predict many of the same things, including aggressiveness, in humans and several other primate species. In an interesting parallel, Carmen Lefevre and her colleagues observed a relationship in capuchin monkeys between facial width and alpha status (a proxy measure of aggressiveness) and a related measure of personality, assertiveness (Lefevre et al., 2014).

## Concluding Remarks

Research on vocal signaling, undertaken in an evolutionary-linguistic context, implies the existence of a huge gulf between humans and other primates. That gulf narrows when one looks at vocal and other cues that are emitted in a social context. That is, humans and other primates are not as different as they have seemed. This conclusion rests not only on similarity at the level of function in social contexts but on the level of shared neural resources.

In this article I have called attention to the meaning or, better, the significance of social vocalizations in humans and other primates, vocalizations that carry personal information. If there is a lesson regarding speech, it is, as F. G. Bailey said, to understand what people are saying we must know who they are and who they are addressing, but much the same is true in the other primates. To understand their actions, we must know things about them, including their rank and reputation, and what they are experiencing at the moment. Without taking full account of these things, we also cannot interpret their behaviors, or them.

This forces us to consider an important question. If we are truly interested in communication, why limit studies of primate vocalization to signals that are broadcast, possibly in response to some attribute of the environment, when, in the case of personal cues, we are confronted with an embarrassment of riches, many in the form of personal information that is critical to a number of different social choices, including collaborators.

Whatever information we humans *think* we’re providing listeners when we speak, a great deal of what we say and do, while speaking, carries information about us, including our reactions to listeners. This sort of information enabled our group-living ancestors to relate, that is, to form relationships, remedy disputes, coordinate activities, and cooperate on important projects, and it continues to do these things today.

As important as information about the emotional and physiological states of others can be, knowing something about the personality and temperament of others enables prediction of their future behaviors, which are of vital significance to the formation of long-term relationships. When physical cues to states and traits are studied across the primate classes, we develop opportunities to witness inter-specific continuity between humans an other primates than is possible when studies are limited to referential functions.

Through cross-disciplinary research that has been carried out in the past two decades, it has become clear that humans communicate many of the things that others need to know about them, as do the other primates. It seems to be the right time for a program of comparative research.

## Author Contributions

The author confirms being the sole contributor of this work and has approved it for publication.

## Conflict of Interest

The author declares that the research was conducted in the absence of any commercial or financial relationships that could be construed as a potential conflict of interest.
